# Identification of co-expression network correlated with different periods of adipogenic and osteogenic differentiation of BMSCs by weighted gene co-expression network analysis (WGCNA)

**DOI:** 10.1186/s12864-021-07584-4

**Published:** 2021-04-10

**Authors:** Yu Liu, Markus Tingart, Sophie Lecouturier, Jianzhang Li, Jörg Eschweiler

**Affiliations:** Department of Orthopaedic Surgery, RWTH Aachen University Clinic, Pauwelsstraße 30, 52074 Aachen, Germany

**Keywords:** Bioinformatics, BMSCs, WGCNA, Osteogenic differentiation, Adipogenic differentiation

## Abstract

**Background:**

The differentiation of bone marrow mesenchymal stem cells is a complex and dynamic process. The gene expression pattern and mechanism of different periods of adipogenic and osteogenic differentiation remain unclear. Additionally, the interaction between these two lineage determination requires further exploration.

**Results:**

Five modules that were most significantly associated with osteogenic or adipogenic differentiation of BMSCs were selected for further investigation. Biological terms (e.g. ribosome biogenesis, TNF-α signalling pathway, glucose import and fatty acid metabolism) along with hub transcription factors (e.g. PPARG and YY1) and hub miRNAs (e.g. hsa-mir-26b-5p) were enriched in different modules. The expression pattern of 6 hub genes, ADIPOQ, FABP4, SLC7A5, SELPLG, BIRC3, and KLHL30 was validated by RT-qPCR. Finally, cell staining experiments extended the findings of bioinformatics analysis.

**Conclusion:**

This study identified the key genes, biological functions, and regulators of each time point of adipogenic and osteogenic differentiation of BMSCs and provided novel evidence and ideas for further research on the differentiation of BMSCs.

**Supplementary Information:**

The online version contains supplementary material available at 10.1186/s12864-021-07584-4.

## Introduction

Bone marrow mesenchymal stem cells (BMSCs), a kind of multipotent stromal cell, have the capability to differentiate into multiple cell types, including adipocyte, osteoblasts, chondrocytes, and myocytes [1, 2]. Dysregulation of its differentiation has been proved to be related to various diseases, such as osteoporosis, which is caused by an imbalance of the differentiation of BMSCs [3, 4]. An increased capacity for adipocyte differentiation but a reduced capacity for osteoblast differentiation raises the susceptibility to brittle fracture for patients who suffer from osteoporosis [5].

It is reported that the lineage determination is a delicate balance between adipogenic and osteogenic differentiation of BMSCs [6]. The master regulator of adipogenesis, peroxisome proliferator-activated receptor γ (PPARγ), and the hub regulator of osteogenesis, runt-related transcription factor 2 (RUNX2), have been demonstrated to suppress each other [7, 8]. However, the differentiation of BMSCs is a dynamic process that contains complex regulation and variation. Little is known about the comprehensive molecular mechanism regarding to the whole procedure of adipogenic and osteogenic differentiation, hindering the development of stem cell therapy.

Several studies have individually investigated the potential biomarkers for osteogenic or adipogenic differentiation based on Differentially Expressed Genes (DEGs) [9, 10]. These studies put forward that certain genes, such as LncRNA MALAT1 and MicroRNA-223 could affect the differentiation of BMSCs. Nevertheless, focusing on a single gene could not demonstrate the mechanism of the whole differentiation process and might neglect some meaningful regulators or pathways throughout the process. Weighted gene co-expression network analysis (WGCNA) is a bioinformatics algorithm method which is designed to identify highly correlated gene clusters and relate them to biological traits [11]. Rather than concentrating on a single gene or an isolated biomarker, WGCNA modularly investigates the co-expressed genes and extracts intramodular hub genes from system networks, increasing the sensitivity to recognise potential worthwhile targets for biological regulations. WGCNA has been widely used for various genomic applications [12, 13].

The presented study comprehensively analysed gene expression patterns through WGCNA at each time point of adipogenic and osteogenic differentiation of BMSCs to investigate the relationship between these two lineages determination. We explored the regulation network for every period of differentiation and validated the analysis results via corresponding cell staining and RT-qPCR experiments. Our study provides valuable research implications for the differentiation of BMSCs and prospective therapeutic targets for clinical stem cell therapy.

## Results

### Data processing and weighted gene co-expression network analysis (WGCNA)

**Fig. 1:**
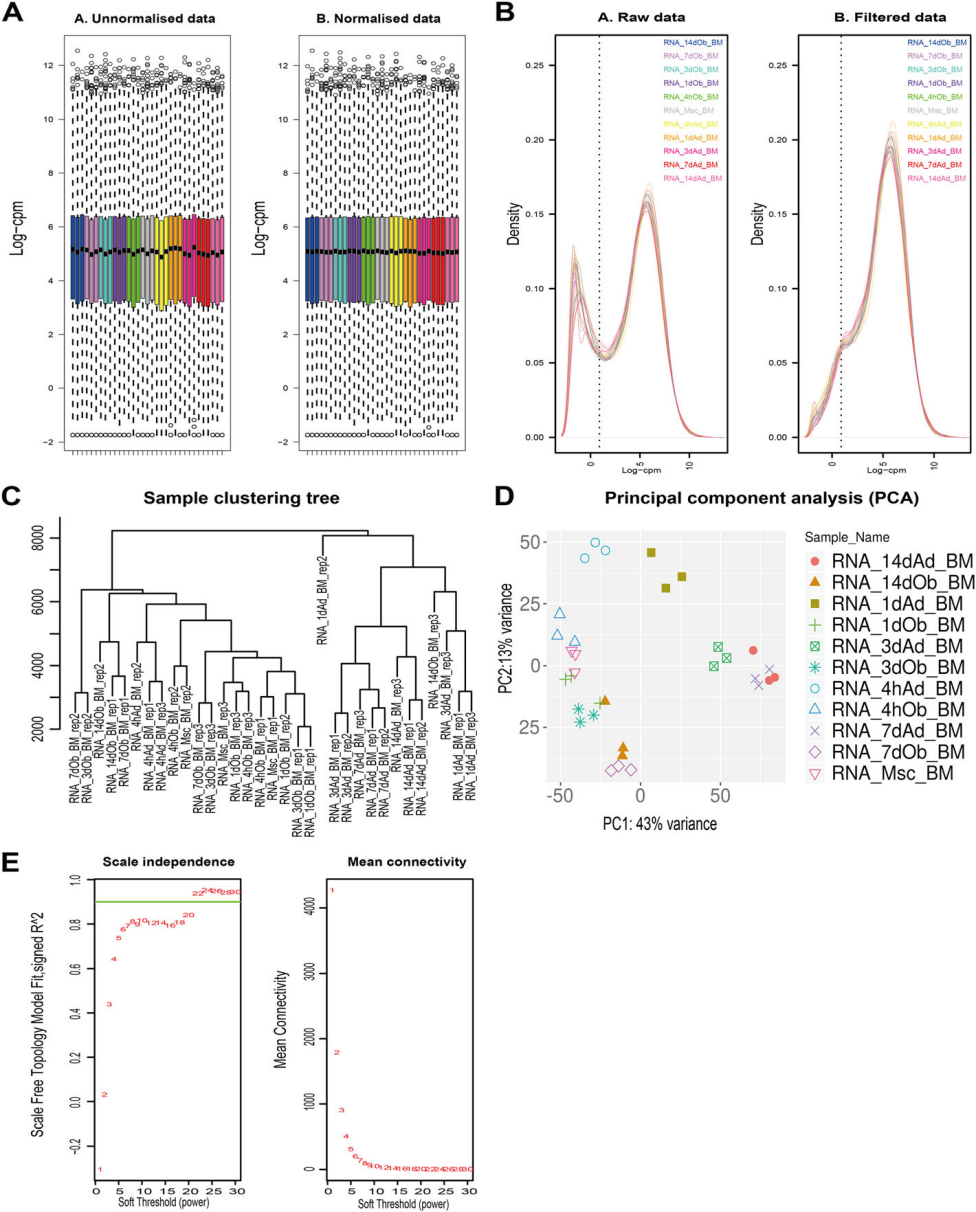
Data processing and procedure of WGCNA. **a** Box plot of sequencing depth analysis for unnormalised and normalised data. Normalised data shows closer sequencing depth. **b** Gene expression level distribution of raw and filtered data. Extremely low expressed genes are filtered. **c**, **d** Hierarchical clustering and PCA to detect outlier sample. **e** Determination of best soft thresholding power for WGCNA. The green line corresponding to 0.9. Twenty-two is selected based on the consideration of both scale independence and mean connectivity. WGCNA, the weighted gene co-expression network analysis. PCA, principal component analysis

Among 170 samples in GSE113253 [6], a total of 33 samples regarding to RNA-Sequencing data of bone marrow-derived human mesenchymal stem cells were selected and underwent data filtration and normalization (Fig. 1a and b). Two samples (RNA_14dob_BM_rep3 and RNA_1dAd_BM_rep2) were excluded because they comparatively differed from other subjects after the outlier detection (Fig. 1c and d). As for the result, 31 samples were included in WGCNA.

The best soft-thresholding, 22, was chosen to construct an approximately scale-free topological overlap matrix (Fig. 1e). Modules were automatically allocated with different colours to distinguish from each other while the genes not clustered were grouped into the grey module (not the grey60 module) (Supplementary file 1 module details). In total, 7329 genes were included in the grey module, accounting for 48.9% of all the analysed genes.

### Identification of differentiation process-related modules

**Fig. 2:**
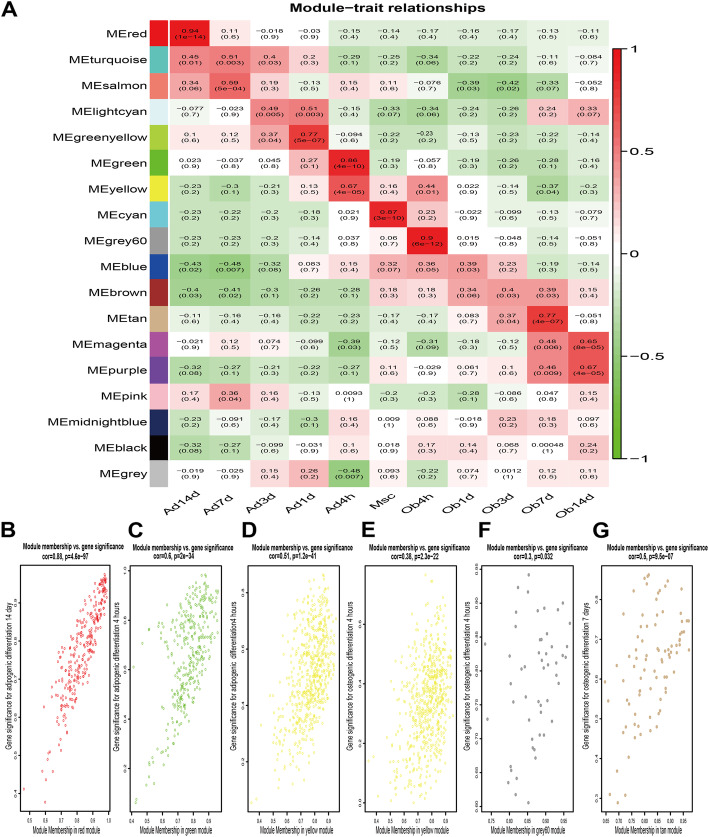
Module-differentiation time point relationships. **a** Heat map of correlation coefficient between modules and differentiation time point. Modules with *p* < 0.05 are considered statistically significant. **b**-**g** Correlation analysis between Gene Significance for certain differentiation time point and Module Membership in the respective module. All the 5 modules show significant correlation (*p* < 0.05) to the corresponding differentiation time point. From (**b**) to (**g**): Ad14d with red module, Ad4h with green module, Ad4h with yellow module, Ob4h with yellow module, Ob4h with grey60 module and Ob7d with tan module. Ad, adipogenic differentiation. Ob, osteogenic differentiation

Correlation between ME and differentiation time point was evaluated through Spearman’s correlation analysis (Fig. 2a). The heatmap showed that 15 modules were significantly associated with one or more differentiation time points (*p* < 0.05). Four modules with the highest correlation coefficient were selected for further research, including red, green, grey60 and tan correlated to adipogenic differentiation 14 days (Ad14d), adipogenic differentiation 4 h (Ad4h), osteogenic differentiation 4 h (Ob4h) and osteogenic differentiation 7 days (Ob7d), respectively. The module yellow was also included as it showed both significant relativity to adipogenic and osteogenic differentiation. The results of the intra-modular analysis demonstrated that the genes in each of the 5 modules were distinctly correlated to the corresponding differentiation time point (Fig. 2b-g), which confirmed the crucial roles of 5 modules in the network of differentiation of BMSCs.

### Biological function annotation of differentiation process-related modules

**Fig. 3:**
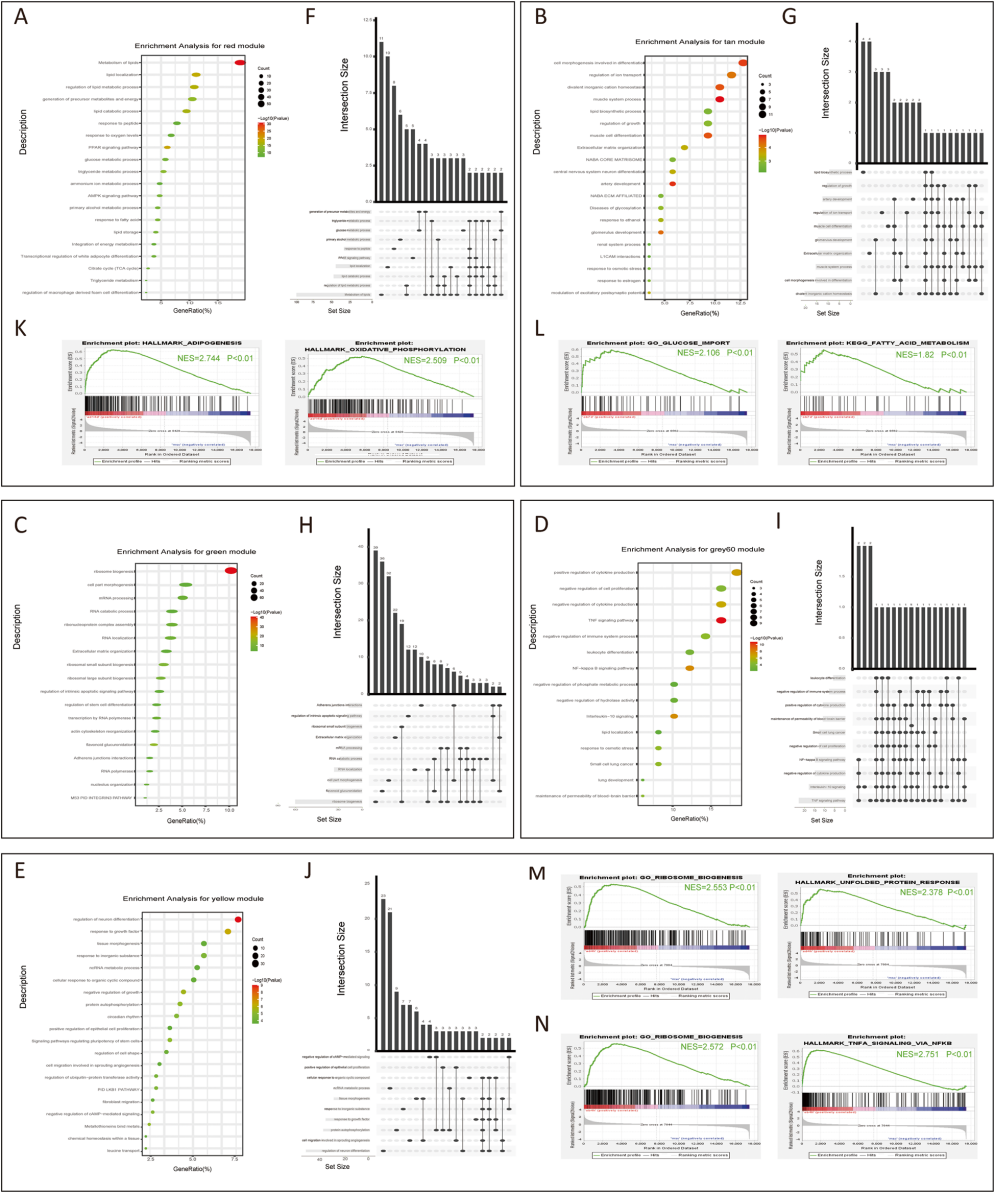
Enrichment analysis for each module and GSEA for various differentiation time of BMSCs. **a**-**e** Dot plot of enriched biological functions in module red, tan, green, grey60 and yellow. The size of dot indicates the number of enriched genes and the colour of dot represents –log10(*P*-value). The X-axis shows the proportion of enriched genes in the overall biological terms. **f**-**j** Upset plot shows overlap of genes across enriched biological functions in module red, tan, green, grey60 and yellow. The grey bar on the bottom left represents the number of genes in each biological term. The black bar on the upper right indicates the number of intersected genes while the dots under the bar show the included biological terms. **k**-**n** GSEA enrichment plots in Ad14d, Ob7d, Ad4h and Ob4h compared to undifferentiated BMSCs. GSEA, gene set enrichment analysis

The enrichment analysis for the biological function of each module was performed through Metascape. The module red, which was related to Ad14d, was involved in the metabolism of lipids, lipid localization, regulation of lipid metabolic process, and PPAR signalling pathway (Fig. 3a and f). This was consistent with the later stage characteristics of adipogenic differentiation. In addition, GSEA revealed that adipogenesis and oxidative phosphorylation were significantly enriched in the 14th day of adipogenic differentiation (Fig. 3k). The most enriched terms of module tan were cell morphogenesis involved in differentiation, muscle system process, divalent inorganic cation homeostasis, and regulation of ion transport (Fig. 3b and g). GSEA indicated the genes in module tan may regulate the mid-term osteogenic differentiation through glucose import and fatty acid metabolism (Fig. 3l).

Ad4h correlated green modules were enriched in ribosome biogenesis and cell part morphogenesis (Fig. 3c and h) which was consistent with the GSEA result (Fig. 3m), indicating the morphology change during the early stage of adipogenic differentiation. Both enrichment analysis (Fig. 3d and i) and GSEA (Fig. 3n) of Ob4h correlated grey60 module showed that regulation of cytokine production and TNF-α signalling pathway play important roles in the early stage of osteogenic differentiation. The module yellow, correlated to both adipogenic and osteogenic differentiation was involved in the regulation of neuron differentiation, response to growth factor, and negative regulation of growth (Fig. 3e and j), suggesting that these terms could possibly affect the direction of differentiation of BMSCs.

### PPI network analysis and hub gene identification

**Fig. 4:**
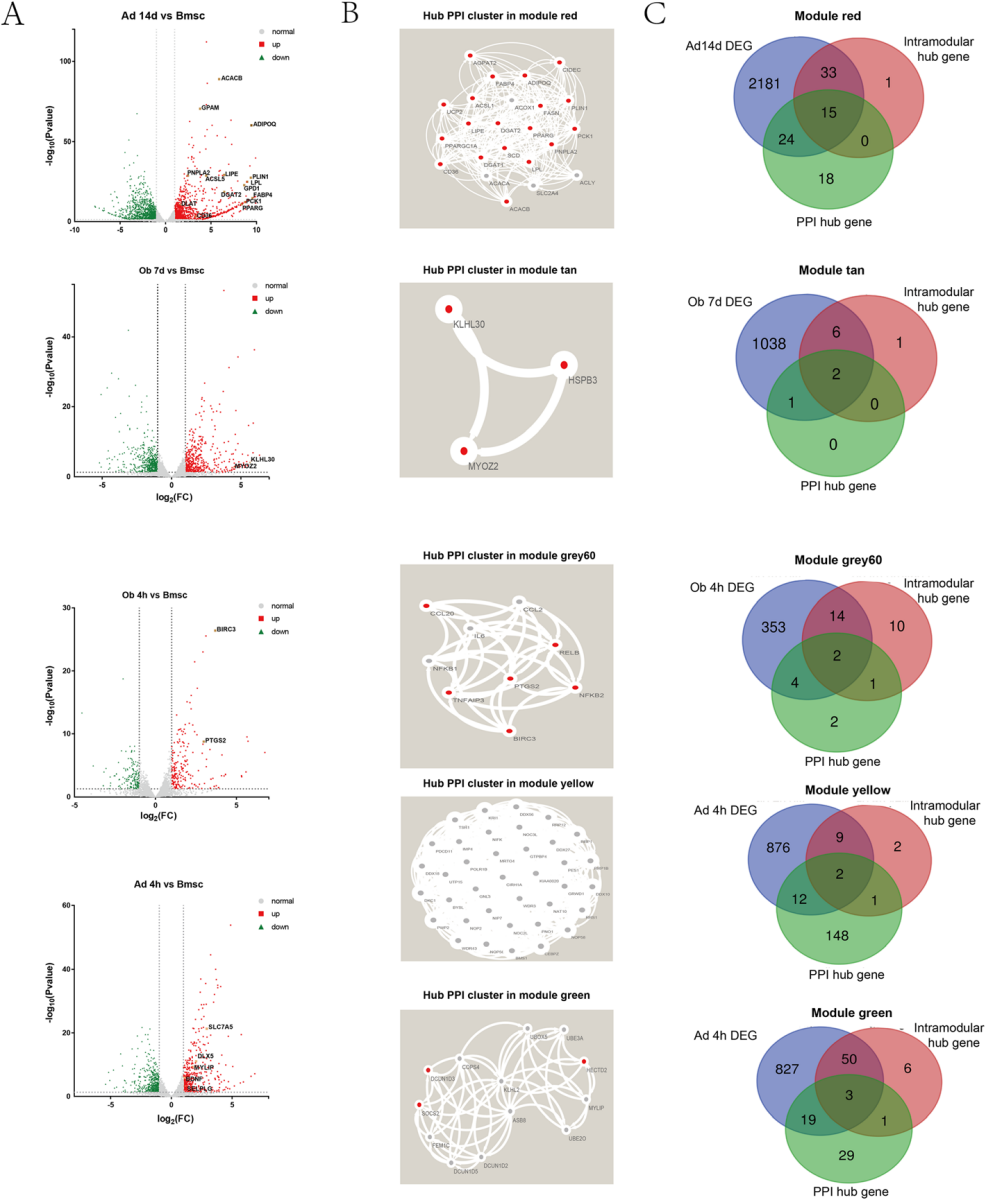
Identification of hub genes. **a** Volcano plot of Differentially Expression Genes (DEGs) at Ad14d, Ob7d, Ob4h and Ad4h compared to undifferentiated BMSC. Red squares indicate upregulated genes (log_2_FC > 1), green triangles indicate downregulated genes (log_2_FC < − 1). Grey dots represent genes that are not statistically different (− 1 < log_2_FC < 1). **b** Typical hub cluster of the PPI network in module red, tan, grey60, yellow and green. Red dots represent upregulated genes at corresponding differentiation time point while grey dots represent genes without statistical differenrence. **c** Venn diagram of overlapped genes between PPI hub cluster genes, DEGs and intra-module hub genes. The overlapped genes are highlighted in fig(A)

To explore the interaction of genes in each module, PPI network was constructed by STRING database (Supplementary materials Fig. S1 A-E). Hub gene clusters with scores above 3 within each PPI network were identified using the Cytoscape MCODE plugin (Fig. 4b).

“EdgeR” package was applied to investigate the DEGs between genes at each differentiation time point and undifferentiated BMSCs with the thresholds of *P*-value < 0.05 and |Fold Change (FC)| > 2.0. In total, 2253 DEGs (1037 upregulated and 1216 downregulated) in Ad14d, 1047 DEGs (472 upregulated and 575 downregulated) in Ob7d, 373 DEGs (208 upregulated and 165 downregulated) in Ob4h and 899 DEGs (416 upregulated and 484 downregulated) in Ad4h were identified (Fig. 4a).

Intra-module hub genes, which possessed high connectivity in each module, were filtered at the threshold of absolute gene significance > 0.8 and absolute intramodular connectivity > 0.8. The intramodular hub genes in each module were listed in Supplementary Materials Table S2. PPI hub cluster genes and DEGs at each differentiation time point were overlapped with high connectivity genes in the respective module. As shown in Fig. 4c, 15 hub genes were overlapped between module red and Ad14d, including ACACB, GPAM, ADIPO1, FABP4, etc.. Two hub genes, KLHL30 and MYOZ2, were overlapped between module tan and Ob7d. Module grey60 and OB4h DEGs shared 2 hub genes: BIRC3 and PTGS2. Module green and Ad4h DEGs shared 3 hub genes: MYLIP, SLC7A5, and DLX5. Module yellow overlapped with Ad4h DEGs by BDNF and SELPLG while it did not overlap with Ob4h DEGs (Table S3).

### Regulation mechanism analysis of differentiation-related modules

**Fig. 5:**
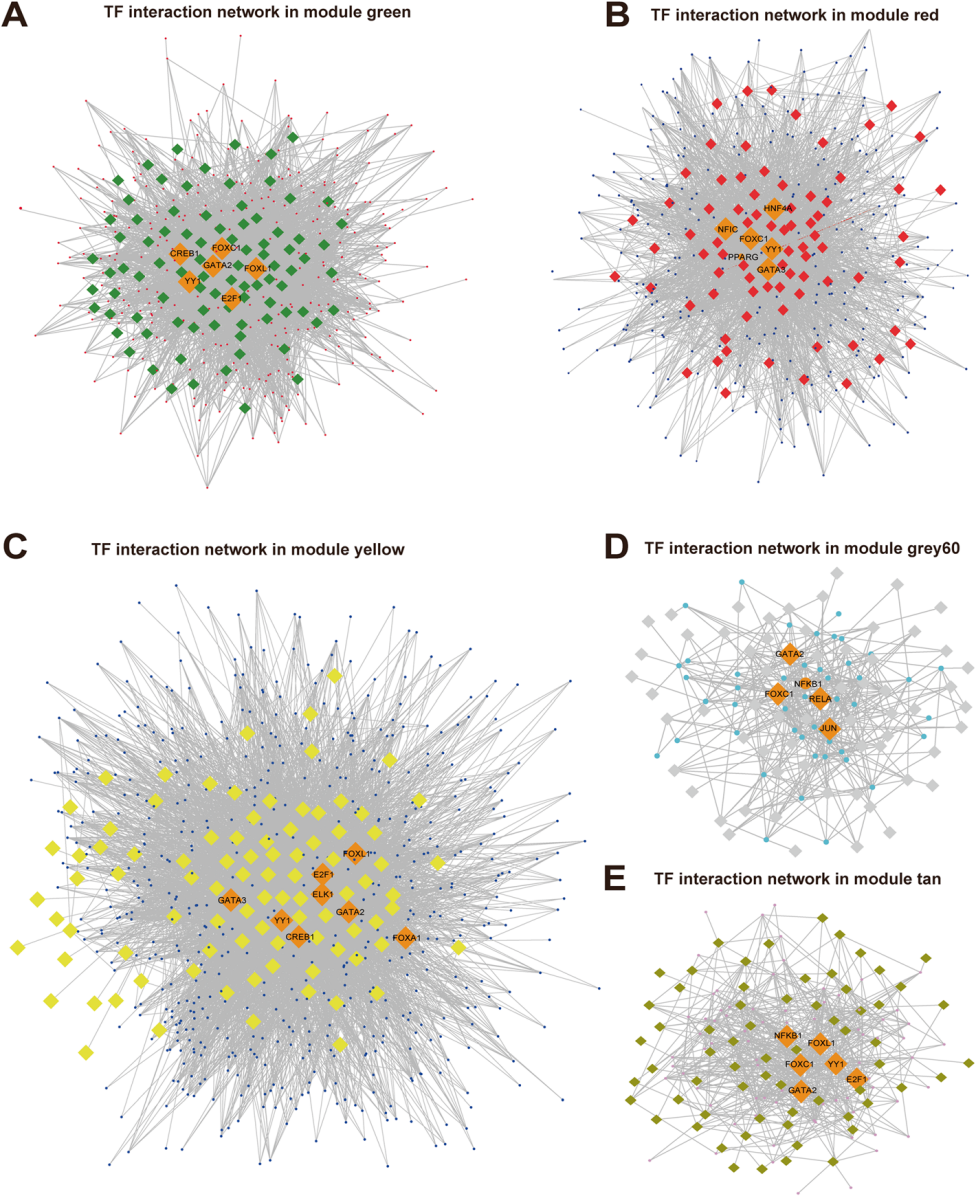
TFs-genes interaction network analysis for each module. **a**-**e** TF-genes interaction network in module green, red, yellow grey60 and tan, respectively. Round dots represent genes in each module and diamonds represent TFs. Hub TFs are highlighted in orange

Given that the modules were consisted of co-expressed genes, they may be regulated by a common mechanism such as transcription factors or miRNAs. The transcription factor binding motifs (TFMFs) enrichment analysis showed that the transcription factor PPARG was the crucial regulator for the red module, which was annotated to the motif transfac_public__M00528. The TFs-genes interaction network analysis for module red indicated that HNF4A, YY1, FOXC1 played important roles in the late stage of adipogenic differentiation as well (Fig. 5b). For module tan, ZNF232, which was annotated to the motif taipale__ZNF232_full_RTGTTAAAYGTAGATTAAG_repr, was the master regulator. GATA2 and YY1 were also found to be key components of the transcription factor network of tan (Fig. 5e), suggesting that they were essential for the regulation of the mid-term of osteogenic differentiation. The transcription factor CREB1 aligned with the motif transfac_pro__M03544 in module green was required for the induction of adipogenic differentiation (Fig. 5a). The transcription factors NFKB1 and RELA which corresponded to motif totransfac_public__M00054 were enriched both in TFMFs and TF-genes interaction analysis in module grey60, revealing their vital roles in the induction of osteogenic differentiation (Fig. 5d). For module yellow, which was correlated to both adipogenic and osteogenic differentiation, the transcription factor ELK1 and its binding motif dbcorrdb__ELK1__ENCSR000EFU_1__m2 were significantly enriched (Fig. 5c). All the enriched motifs and their high confidence transcription factors were shown in Supplemental Digital Content (SDC1-SDC5).

**Fig. 6:**
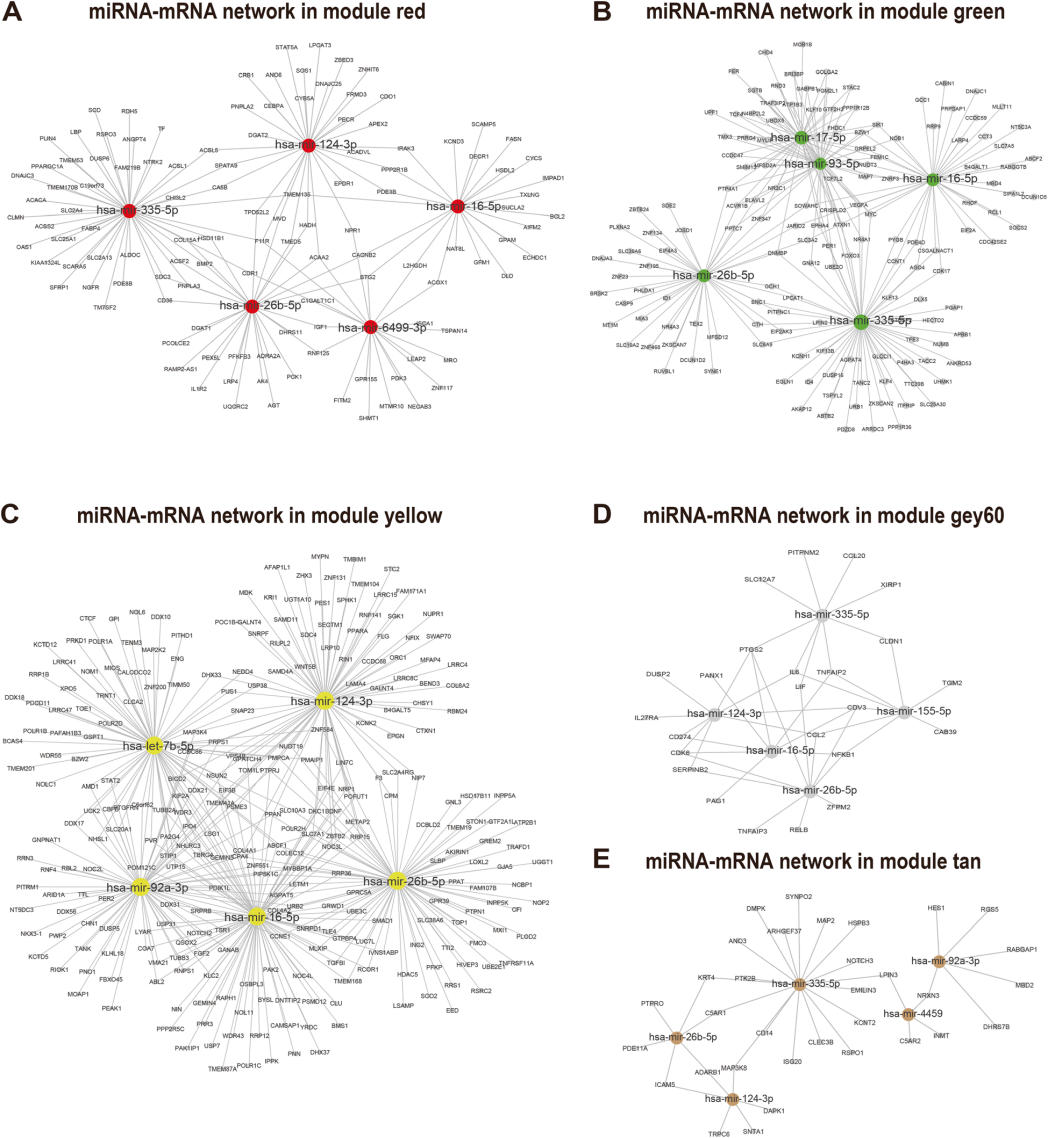
MiRNAs-mRNAs interaction network analysis for each module. **a**-**e** MiRNAs-mRNAs interaction network in module red, green, yellow, grey60 and tan, respectively. The top5 miRNAs regulated the greatest number of genes are highlighted in the colour corresponding to each module. The smaller grey dots represent the mRNAs

MiRNAs are another fundamental regulator through recognition of cognate sequence which participate in transcriptional, translational, or epigenetic processes. Consequently, we constructed a miRNAs-mRNAs interaction network based on experimentally validated miRNA-target pairs in Tarbase v8.0. The top5 miRNAs regulating the greatest number of the genes were highlighted in each module (Fig. 6). Hsa-mir-26b-5p functioned in all 5 modules, indicating its pivotal influence in the differentiation of BMSCs. Other hub miRNAs, including hsa-mir-335-5p, hsa-mir-16-5p, and has-mir-124-3p, were also regulated in both adipogenic differentiation-related and osteogenic differentiation-related modules, suggesting the intimate interaction between two directions of the differentiation of BMSCs. For further exploration of the regulatory relationship between TFs, miRNAs and mRNAs, the TF-miRNA-mRNA coregulatory network for each module was constructed. In addition, miRNA-mRNA pairs which shared the same targeted TF were extracted from the whole network (Fig. S2 A-E). Detailed information of all the pairs was provided in the supplementary file 2, 3 and 4.

### Differentiation-related staining and hub gene validation

BMSCs demonstrated positive expression of the surface markers CD73, CD90 and CD105 and negative expression of the surface markers CD19, CD34 and CD45 (Fig. S3).

**Fig. 7:**
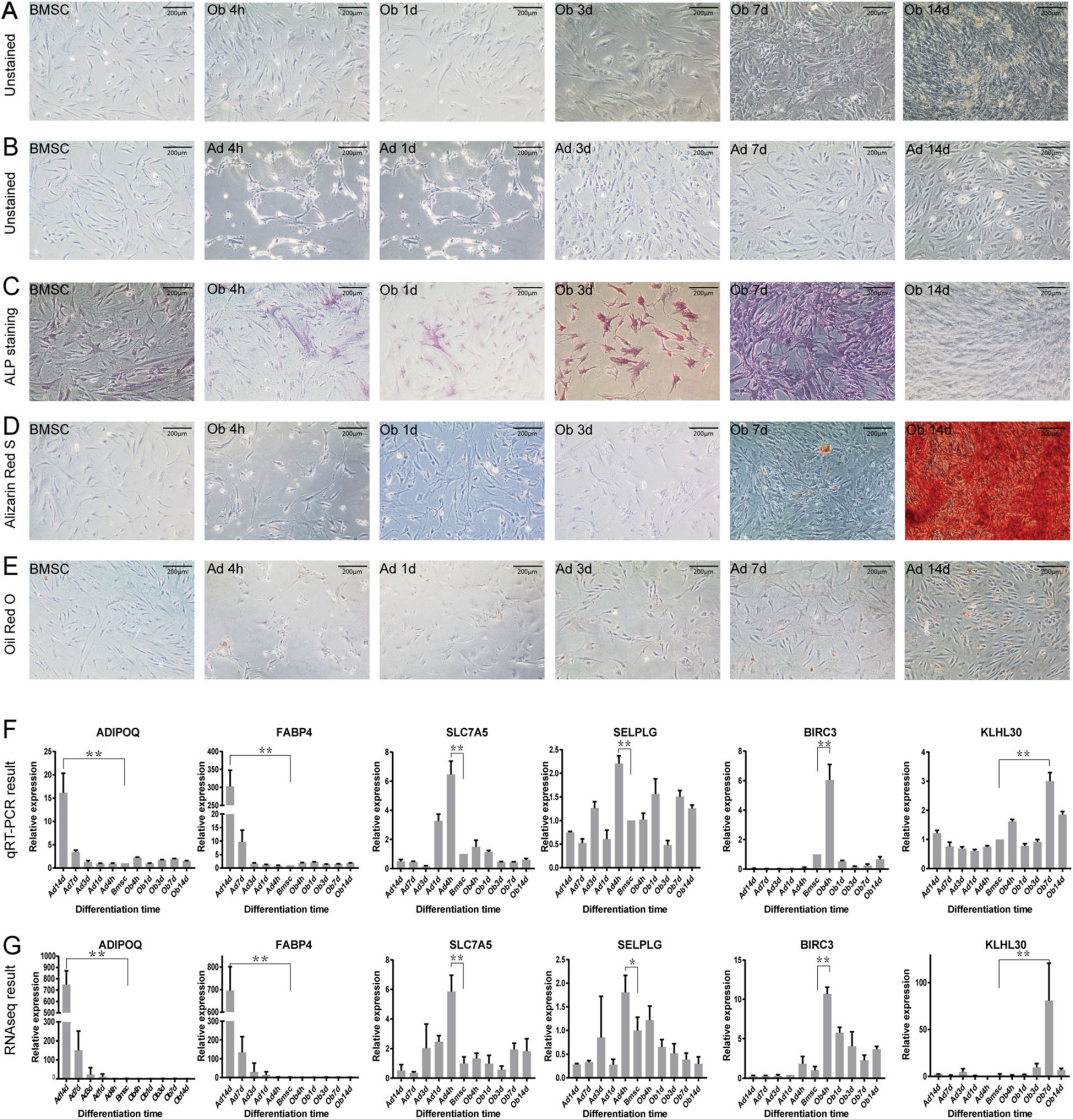
adipogenic and osteogenic differentiation of BMSCs and hub genes expression validation. **a** Unstained BMSCs and osteogenic differentiated BMSCs from 4 h to 14d (10x). **b** Unstained BMSCs and adipogenic differentiated BMSCs from 4 h to 14d (10x). **c** ALP stained BMSCs and osteogenic differentiated BMSCs from 4 h to 14d (10x). **d** Alizarin Red S stained BMSCs and osteogenic differentiated BMSCs from 4 h to 14d (10x). **e** Oil Red O stained BMSCs and adipogenic differentiated BMSCs from 4 h to 14d (10x). **f** The relative expression level of selected hub genes based on RT-qPCR. **g** The relative expression level of hub genes based on RNA sequencing in dataset GSE113253. *p < 0.05; **p < 0.01

As shown in Fig. 7b, the morphology of BMSCs started to gradually transform from arborisation into round after 4 h of adipogenic differentiation, which was coincident with the biological function enrichment analysis of module green. Lip droplets existed from the 7th day of adipogenic differentiation and continuously accumulated until the 14th day. The result of Oil Red O staining also illustrated the transformation process of adipogenic differentiation (Fig. 7e).

For osteogenic differentiation, BMSCs initiated the morphological change and mineralization deposit after 7 days (Fig. 7a). Alizarin Red S staining demonstrated that mineralization accumulation increased rapidly from the 7th day to the 14th day of osteogenic differentiation (Fig. 7d). However, ALP rose from 4 h after osteogenic differentiation and reached the peak at the 7th day (Fig. 7c). After that, the level of ALP began to decrease and significantly downregulated at the 14th day. Such phenomenon revealed that the 7th day was a crucial turning point of osteogenic differentiation, which was corresponding to our WGCNA results.

Finally, we validated the hub gene expression levels during the differentiation of BMSCs. Six hub genes from 5 modules were selected, including ADIPOQ, FABP4, SLC7A5, SELPLG, BIRC3, and KLHL30. All the hub genes showed significant overexpression at their respective differentiation time point. Their expression trend was consistent with the RNA sequencing results in dataset GSE113253 (Fig. 7f and g), which certified that the results of this study were reliable and accurate.

## Discussion

Recent studies demonstrated that the osteogenic and adipogenic lineages could alternate during cell differentiation, indicating the subtle and complicate relation between them [14, 15]. The underlying mechanism during the differentiation process remains unclear. In this study, we identified several modules that highly correlated to diverse stages of differentiation and performed enrichment analysis for each module. Hub genes and crucial regulation factors, such as miRNAs and TFs, were identified from the networks.

The grey60 module was significantly associated with OB4h. Enrichment analysis and GSEA for grey60 module revealed the significance of the TNF-α via NF-κB signalling pathway in the early stage of osteogenic differentiation. Interestingly, TNF-α was widely accepted as an inhibitor of osteogenic differentiation and osteogenesis among previous studies [16, 17]. However, some recently contradictory findings suggested the paradoxical effects of TNF-α in the regulation of bone homeostasis [18]. Daniele et al. demonstrated that the effect of TNF-α on osteogenic differentiation was dose-dependent [19]. A low concentration of TNF-α showed enhanced osteogenic differentiation of BMSCs while a high concentration brought about an opposite result. Moreover, Huang et al. pointed out that treatment time also affected the function of TNF-α in osteogenic differentiation [20]. In addition, the hub gene BIRC3 and the hub transcription factor RELA derived from our grey60 module could be activated by NF-κB through the TNF-α signalling pathway [21]. The expression of BIRC3, as shown in our RT-qPCR results, considerably increased after 4 h of osteogenic differentiation and decreased immediately afterward, which was perfectly consistent with the dual effects of TNF-α in osteogenic differentiation.

Mid-term osteogenic differentiation was correlated to the module tan, in which transcription factors FOXC1, FOXL1, YY1 and GATA2, and miRNAs hsa-mir-335-5p, hsa-mir-92a-3p, hsa-mir-16b-5p, hsa-mir-4459, and hsa-mir-124-3p were master regulators. KLHL30 and MYOZ2 were overlapped between Ob7d DEGs, intramodular hub genes, and hub PPI cluster. KLHL30 is a protein coding gene which contains a bric-a-brac domain. It was reported that such genes could be related to the dynamic changes in chromosomes [22]. However, research on their functions in osteogenesis is still missing. MYOZ2 belongs to a family of sarcomeric proteins that bind to calcineurin, a kind of phosphatase. These family members are important for regulation of calcineurin signalling in cardiac and skeletal muscle cells [23], which could also be necessary for the differentiation and mineralization of osteoblasts. Cell morphogenesis involved in differentiation was enriched in this module, which was also observed under a microscope during our differentiation induction. The 7th day of osteogenic differentiation was a turning point for ALP production and mineralization deposit. Our result was consistent with the finding that ALP activity was a very early osteogenic marker which was significantly upregulated in mesenchymal cells prior to osteoid or mineral deposition [24, 25]. This expression pattern of ALP has also been reported in the osteogenic differentiation process of cells derived from other sources [26]. Jaiswal et al. [27], demonstrated that the expression of ALP peaks was an indication of the presence of osteoprogenitor cells which was followed by an active expression of osteocalcin, a marker for the mineralization stage. The differentiation of BMSCs in vitro is extremely dependent on the cell source, culture conditions and other factors. Many other studies reported that ALP peak existed on the 14th day of differentiation and the mineralization appeared until 21st - 28th day [28, 29]. As a result, the mechanism of this time-related expression pattern of ALP during osteogenic differentiation remains to be investigated. GSEA results demonstrated that glucose import and fatty acid metabolism were significantly enriched in Ob7d compared to undifferentiated BMSCs. Glucose has been recognised to be an essential nutrient for osteoblasts and many other studies have already focused on the glutamine metabolism in bone homeostasis [30, 31]. Fatty acids also exhibited close implication in osteogenesis [32]. Our research confirmed the importance of nutrient metabolism and proposed a possible time point for them to function during the process of osteogenic differentiation.

According to the results of cell staining and differentiation induction, adipogenesis experienced considerably more obvious and faster changes than osteogenesis at the very early stage of differentiation. The sample cluster analysis and the PCA supported that the transcriptome of osteoblasts clustered closer to BMSCs than that of adipocytes after 4 h of differentiation. The analysis of module green, which was highly related to Ad4h, showed that ribosome biogenesis was significantly enriched. Besides, ribosome biogenesis was both enriched in Ob4h and Ad4h with GSEA, indicating its decisive role in the fate of cell differentiation. Our conclusion was in agreement with previous studies on other stem cells [33, 34]. Three hub genes, SLC7A5, MYLIP, and DLX5 were overlapped between module green and DEGs of Ad4h. SLC7A5 (also known as LAT1), a sodium-independent Neutral Amino Acid transporter, has been widely investigated in various cancer cells [35]. Recently, Beaumatin et al., demonstrated that SLC7A5 could affect cellular growth, metabolic homeostasis, and differentiation by activating mTORC1 through DRAM-1, which was a key regulator for controlling adipocyte signalling and differentiation [36]. Another overlapped hub gene, MYLIP, had also been reported to be related with lipid absorption and metabolism. It was induced by sterol-responsive nuclear receptors (LXRs) to ubiquitinate LDLR for degradation, which limited the uptake of lipoprotein-derived cholesterol [37]. Moreover, transcription factor CREB1, annotated to motif transfac_pro__M03544 and transcription factor ATF1, annotated to motif transfac_pro__M07034 were identified from the TFMFs enrichment analysis for module green. Further research on these hub genes and hub transcription factors are required for uncovering the mechanism of early adipogenic differentiation of BMSCs.

Compared to the early stage of adipogenic differentiation, the late period of adipogenesis mainly focuses on dealing with lipids. The top activated biological terms in module red were metabolism of lipids, lipid localization, lipid catabolic process, and regulation of lipid metabolic process. The upset plot showed many genes were overlapped in these terms. A total of 15 hub genes were recognised from module red, among which, PPARG, ADIPOQ, LPL, and FABP4 were already well-known biomarkers of adipocytes [38, 39]. Other hub genes in module red, e.g. LIPE, had intimate interactions with these validated biomarkers, suggesting their unignorable position during the late stage of adipogenesis. LIPE, a kind of lipase encoding gene, has been reported to be involved in various lipid metabolism-related syndrome [40, 41]. However, few studies have reported the relationship between LIPE and adipogenic differentiation. Our TFMFs enrichment analysis of red module also obtained some motifs that aligned with transcription factor PPARG, including transfac_public__M00528, cisbp__M3785, and cisbp__M6433. These enriched motifs along with the hub miRNAs in the miRNAs-mRNAs network in module red, including hsa-mir-335-5p, hsa-mir-124-3p, hsa-mir-26-5p, hsa-mir-6499-3p, hsa-mir-16-5p, could be the master regulators of the late stage of adipogenic differentiation.

Module yellow was the only module that was significantly correlated to both adipogenic and osteogenic differentiation, which supported the viewpoint that these two lineage determination interacted with each other closely. Many common genes were involved in both differentiation and affected the destiny of BMSCs. As a result, the enriched terms in module yellow, for example, regulation of neuron differentiation, response to growth factor, and negative regulation of growth could function in early-stage differentiation of BMSCs. Nevertheless, hub genes, SELPLG and BDNF, were only overlapped between module yellow and DEGs of Ad4h, but not DEGs of Ob4h. This phenomenon may result from the situation we mentioned above that adipogenesis changed earlier and more discernible than osteogenesis. SELPLG, identified as a myeloid cell intrinsic factor, was reported critical for cell migration and chemotaxis [42]. In addition, SELPLG could serve as a signal transduction receptor to trigger intracellular signal events, which could be important for both adipogenic and osteogenic differentiation of BMSCs [43]. Another hub gene, BDNF, was proved to play dual effect on adipogenesis, which was corresponded to the duplex function of module yellow. F. Bernhard et al. [44], reported significant downregulated expression of BDNF with induction of adipocyte differentiation while BDNF expression was increased in adipose-derived stem cells treated with a neurogenic induction protocol [45]. This may indicate the effect of module yellow on the commitment of cell lineage determination.

All the hub genes screened from the 5 modules represented the crucial biological functions during different time points of the differentiation of BMSCs. In general, adipogenic differentiation related genes functioned in the process of lipid absorption and metabolism, adipocyte signalling and lipase regulation while osteogenic differentiation related genes were more related to calcium and phosphorus metabolism and mineralization deposit. In addition, basic nutrition related genes and signal transduction receptor were both necessary for differentiation of BMSCs. For the regulation network of differentiation, we found many hub transcription factors and hub miRNAs existed in more than one module, such as YY1, GATA2, E2F1, hsa-mir-26b-5p, hsa-mir-335-5p, and hsa-mir-16-5p, suggesting that these master regulators could be active in the whole process of differentiation. Although, constrained by the length of the article and practical situation, some of the findings could not be explained and examined in detail, there was no doubt that our study supplied some potential research targets for further investigation in the differentiation of BMSCs despite the limitations.

## Conclusion

Taken together, we applied WGCNA for exploring transcriptome data of various time points of differentiation of BMSCs and identified 5 modules that were significantly correlated to different stages of osteogenic or adipogenic differentiation. The pivotal biological terms, hub genes, and master regulators for each time point of differentiation were predicted through bioinformatics analysis. Meanwhile, cell experiments were conducted as the verification of bioinformatics analysis. We are looking forward to providing novel evidences and ideas for further research on the differentiation of BMSCs.

## Materials and methods

### Data acquisition and pre-processing

The publicly available dataset GSE113253, which contained 170 samples of various mesenchymal stem cells, was downloaded from Gene Expression Omnibus (GEO). Among all the samples, 33 total RNA-sequencing data of bone marrow-derived human mesenchymal stem cells (BMSCs) were selected for further analysis. First, ‘filterByExpr’ in the ‘edgeR’ package in R 3.6.3 (R Core Team, 2020, https://www.r-project.org) was applied to filter the low-expression raw data and ‘calcNormFactors’ was applied to normalise the sequencing depth difference of all the samples. R is a free software environment for statistical computing and graphics. Furthermore, we conducted hierarchical clustering and Principal Component Analysis (PCA) to eliminate outlier samples accordingly. Finally, 31 samples (14 osteogenic BMSCs, 14 adipogenic BMSCs, and 3 undifferentiated BMSCs) were included in our analysis.

### Construction of weighted gene co-expression network

We constructed the co-expression network through “WGCNA” package under R environment (https://horvath.genetics.ucla.edu/html/CoexpressionNetwork/Rpackages/WGCNA/). Firstly, the gene expression file and trait file were transformed into an appropriate format and the soft thresholding power (β value) was filtered based on the calculation of scale-free topological fit index and mean connectivity. The best β value was confirmed with a scale-free fit index bigger than 0.85 as well as the highest mean connectivity by performing a gradient test from 1 to 30. After that, the topological overlap matrix (TOM) was constructed by calculating the topological overlap between pairwise genes, and hierarchical clustering analysis was performed. The co-expression relationships among different modules were analysed and modules with high similarity were merged at the threshold of 0.25.

### Module-trait correlation analysis and identification of interesting modules

To excavate interested modules which were highly related to the differentiation of BMSCs, correlation analysis between each module and different time points of adipogenic or osteogenic differentiation were conducted. This relationship was determined by Spearman’s correlation coefficient between module eigengene (ME, the major component of gene expressions in a module) and differentiation traits. Modules which had significant correlations with differentiation traits were selected for further validation.

Subsequently, gene significance (GS) and module membership (MM) were used for intramodular analysis. GS is the relationship between gene expression level and differentiation trait while MM represents the association between gene expression profile and ME of a given module. The modules containing genes with a significant correlation between GS and MM were considered meaningful.

### Enrichment analysis for biological function and gene set enrichment analysis

To investigate the biological function of the differentiation-related modules, enrichment analysis such as Gene Ontology (GO), Kyoto Encyclopedia of Genes and Genomes (KEGG) pathway [46–48], hallmark gene set, etc., was performed through Metascape (https://metascape.org/gp/), which is a comprehensive gene annotation and analysis resource [49]. Terms with a *P*-value < 0.01, a minimum count of 3, and an enrichment factor > 1.5 were collected and grouped into clusters based on their membership similarities.

For further verification of the function of the modules during the respective differentiation process, we conducted the Gene Set Enrichment Analysis (GSEA) for each time point of differentiation. The gene expression pattern in certain differentiation point was compared to the undifferentiated BMSCs. The criteria for statistical significance were set as *p* < 0.01 and FDR < 0.25.

### Construction of protein-protein interactions network and hub gene identification

To study the potential mechanism under the differentiation of BMSCs, we constructed a Protein-Protein Interactions (PPI) network through the Search Tool Retrieval of Interacting Genes / Proteins (STRING) database, which is a trustworthy online database that can predict the physical and functional association between known and predicted PPI.

The MCODE package in Cytoscape (version 3.8, https://cytoscape.org/) was applied to identify the hub proteins which have a high degree of connectivity in the whole network. Meanwhile, we calculated the intra-modular connectivity and significance for all genes in the interested module. The genes with absolute gene significance > 0.8 and absolute intramodular connectivity > 0.8 were screened to compare with the hub genes from the PPI network. Moreover, the” edgeR” package was used to determine the DEGs between differentiated cells and undifferentiated BMSCs at each time point. The overlapped genes between PPI hub protein, intramodular hub genes, and DEGs were deemed to be key candidate genes that may regulate the differentiation of BMSCs.

### Transcription factors and target-miRNA interaction analysis for interested modules

To recognise the crucial regulation factors for the differentiation of BMSC, we performed the enrichment analysis of transcription factor binding motifs (TFBMs) through the RcisTarget package under the R environment. The databases, hg19-tss-centered-10 kb-7species.mc9nr (species = Homo sapiens, genome = hg19, distance = 10 kb around the transcription start site (TSS), number of orthologous species (nOrt) =7, motif collection = Version 9 (mc9nr): 24453 motifs), were utilized to analyse the gene list of each interested module. The significant motifs based on the Normalized Enrichment Score (Jaiswal et al.) and the genes with the best enrichment for each motif were outputted. Additionally, the TFs-genes interaction network was constructed by NetworkAnalyst (https://www.networkanalyst.ca/), which is a comprehensive network visual analytics platform for gene expression analysis.

The Tarbase v.8, a decade-long collection of experimentally supported miRNA-gene interactions, was used to seek out the potential miRNAs that can target the mRNAs within interested modules [50]. To improve the reliability of the predicted miRNAs, only the experimentally validated mRNAs-miRNAs pairs were included. Based on the number of targeted mRNAs, the top 5 miRNAs were extracted and the miRNA-mRNA network was visualized by Cytoscape 3.8. Meanwhile, top 5 miRNAs targeted TFs were predicted through RegNetwork (http://www.regnetworkweb.org/). Based on the interaction between each pair of miRNA-mRNA, TF-miRNA and TF-mRNA, the TF-miRNA-mRNA coregulatory network was constructed and visualized by Cytoscape 3.8 as well.

### Cell culture and differentiation

BMSCs extracted from the human femoral head [51] were cultivated in Dulbecco’s Modified Eagle Medium (DMEM; 21,885–025; Gibco, Germany) supplemented with 10% fetal bovine serum (FBS; P30–3702; PAN BIOTECH, Germany) and 1% penicillin-streptomycin (15140–148; Gibco, Germany) at 37 °C in an incubator with 5% CO_2_. The culture medium was changed every 3 days. The immunophenotypes of cells were determined by specific antibodies through flow cytometer. Passage 2–4 of BMSCs were used for differentiation for 2 weeks. 4*10^4^ and 2*10^5^ cell per well were seeded in 6 well plate for osteogenic and adipogenic differentiation, respectively. To induce osteogenic differentiation, the culture medium was replaced by DMEM with 100 nM dexamethasone (D2915-100MG; Sigma, Germany), 10 nM sodium-β glycerophosphate (G5422-25G; Sigma, Germany), 0.05 mM L-ascorbic acid (A8960-5G; Sigma, Germany), 10% FBS and 1% penicillin-streptomycin. The adipogenic differentiation was induced by DMEM with 1 μM dexamethasone, 0.5 mM 3-isobutyl-1-methylxanthine (IBMX; I5879; Sigma, Germany), 200 μM indomethacin (I7378-5G; Sigma, Germany), 10 μM insulin (I9278; Sigma, Germany), 10% FBS and 1% penicillin-streptomycin. The medium was also changed every 3 days.

### ALP staining, alizarin red S staining, and oil red O staining

After differentiation for 4 h, 1 day, 3 days, 7 days and 14 days, the cells were fixed with 4% PFA for 30 min at room temperature and washed with PBS twice. After that, cells were stained with ALP staining kit (ab242286; Abcam, Germany) to detect the change of Alkaline Phosphatase, Alizarin Red S (C.I. 58,005; ROTH, Germany) to observe the mineralization, and Oil red O working solution (O0625-25G; Sigma, Germany) to inspect the accumulation of lipids, according to the manufacturing introduction, respectively. The stained cells were observed and recorded by microscopic analysis.

### RNA extraction and real-time quantitative PCR (RT-qPCR)

Total RNA of BMSCs and differentiated cells was extracted with RNA-Solv Reagent (R6830–02; Omega, Germany) and was reverse transcribed into complementary DNA by High-Capacity RNA-to-cDNA™ Kit (4,387,406; Thermo Fisher Scientific, Germany) according to the manufacture instruction. RT-qPCR was performed on a 7300 Real-Time PCR system (Applied Biosystems, Germany) using SYBR™ Green Mastermix (4,385,612; Thermo Fisher Scientific, Germany). The results were normalised to the expression level of GAPDH and the relative expression levels of each gene were calculated by the 2^-ΔΔCt^ method. The sequences of primers are listed in Supplementary materials (Table S1).

## Supplementary Information

**Additional file 1.**

**Additional file 2.**

**Additional file 3.**

**Additional file 4.**

**Additional file 5.**

## Data Availability

The dataset analysed during the current study are available in the GEO repository, https://www.ncbi.nlm.nih.gov/geo/query/acc.cgi?acc=GSE113253

## Abbreviations

BMSCs: Bone marrow mesenchymal stem cells
WGCNA: Weighted gene co-expression network analysis
Ad: Adipogenic differentiation
ALP: Alkaline Phosphatase
DEGs: Differentially Expressed Genes
DME: Dulbecco’s Modified Eagle Medium
FBS: Fetal bovine serum
GEO: Gene Expression Omnibus
GO: Gene Ontology
GS: Gene significance
GSEA: Gene Set Enrichment Analysis
KEGG: Kyoto Encyclopedia of Genes and Genomes
MM: Module membership
NES: Normalized Enrichment Score
O: Osteogenic differentiation
PCA: Principal Component Analysis
PPI: Protein-Protein Interactions
RT-qPCR: Real-Time quantitative PCR
TFs: Transcription factors
TFBMs: Transcription factor binding motifs
TOM: Topological overlap matrix
