# Supplementary material for: Identification of co-expression network correlated with different periods of adipogenic and osteogenic differentiation of BMSCs by weighted gene co-expression network analysis (WGCNA)
Source: BMC Genomics. 2021 Apr 10;22:254. doi: 10.1186/s12864-021-07584-4 (PMC8035768; doi:10.1186/s12864-021-07584-4)
Supplement: Supplementary file 5 — Additional file 5. [file 12864_2021_7584_MOESM5_ESM.zip › supplementary materials revision.pdf]

Identification of co-expression network correlated with different periods of adipogenic and osteogenic differentiation of BMSCs by Weighted gene co-expression network analysis (WGCNA)

Yu Liu [a*], Markus Tingart [a], Sophie Lecouturier [a], Jianzhang Li [a], Jörg Eschweiler [a]

[a] Department of Orthopaedic Surgery, RWTH Aachen University Clinic, 52074 Aachen, Germany

***CORRESPONDING AUTHOR:**

Yu Liu, MD

Department of Orthopaedic Surgery,

RWTH Aachen University Clinic

Pauwelsstraße 30

52074 Aachen, Germany

Phone: +49 (0) 241 80 80470

E-mail: yliu@ukaachen.de

Email addresses: Yu Liu: yliu@ukaachen.de; Markus Tingart: mtingart@ukaachen.de; Sophie Lecouturier: slecouturier@ukaachen.de; Jianzhang Li: jli@ukaachen.de; Jörg Eschweiler: joeschweiler@ukaachen.de

Supplementary Materials

1.Supplementary Table

Gene Symbol		Primer Sequence
GAPDH	Forward:	ACAACTTTGGTATCGTGGAAGG
	Reverse:	GCCATCACGCCACAGTTTC
ADIPOQ	Forward:	AACATGCCCATTCGCTTTACC
	Reverse:	TAGGCAAAGTAGTACAGCCCA
FABP4	Forward:	ATGAAAGAAGTAGGAGTGGGCT
	Reverse:	GCATTCCACCACCAGTTTATCA
SLC7A5	Forward:	GACCGTCCTGTGTCAGAAGC
	Reverse:	TCAGTTGAGGGATGAGATTCGTA
SELPLG	Forward:	AGGAGATAAGATGGCTGGTGC
	Reverse:	AGTGGTGTCAGTGCTGTTCC
BIRC3	Forward:	TGGGAACCGAAGGATAATGC
	Reverse:	GCTGTTCAAGTAGATGAGGGTAA
KLHL30	Forward:	TCTATGTCACAGGTGGCTCTC
	Reverse:	TGGTGCCGCCGATAACG

Table S1: Primer Sequence.

Module	Hub Genes
**Red**	GPAM, PNPLA2, DLAT, ACACB, ZNF117, VKORC1L1, HADH, ERV31, ADRA2A, A2M, ADRBK2, LRRC8B, NR1H3, ADIPOQ, ACSL5, LIPE, GYG2, LPL, ACVR1C, TM7SF2, ADIPOQAS1, PLIN1, SYN2, AOC2, GPD1, SLC19A3, CHI3L2, FABP4, DGAT2, PLIN4, CHCHD10, KLB, AOC3, ABCD2, PRKAR2B, SEMA3G, CD36, CEBPA, RGAG1, BBOX1, NPR1, FAM213A, ITIH5, PCK1, PPARG, FABP5, TUSC5, PALMD, THRSP
**Green**	AKAP12, SLC7A5, ITPRIP, DNMBP, ST3GAL1, SLC19A2, FAM46A, PHLDA1, PTP4A1, MEDAG, CTDSP2, EPHA4, ZFAND5, FAM167A, RANBP2, ATP1B3, PACSIN2, RIMKLB, SIX2, ZNRF3, PGM2L1, PHF20, CMIP, DUSP4, MYLIP, DLX5, MAP3K4, CDK17, PIK3R3, EGLN1, CSNK1D, SYAP1, PPTC7, GABPB1, KLHL26, JARID2, GZF1, EIF2AK3, N4BP2L2, EIF4A3, ZNF28, SIK1, SOWAHC, LINC01128, DNMBP-AS1, ZNF347, SS18L1, ABTB2, OSGIN1, CAMKK1, ERN1, SLC22A4, OTUD3, CREM, LOC101928371, FAM49A, BMP8A, MUC13, USP2, C2orf66
**Tan**	NATD1, C5AR1, PLXDC1, CD14, INMT, MYOZ2, MIR145, C1QTNF7, KLHL30
**Grey60**	PHLPP2, BIRC3, TNFAIP2, RBM12, NFKB1, PANX1, CAB39, KRT34, PTGS2, FAM101A, ZNF664-FAM101A, PSPC1, CD83, PITPNM2, RUNDC1, PAG1, FOSB, IL27RA, METTL7B, EPPK1, SERPINB2, STRA6, XIRP1, ISLR2, ELFN2, FAM196A, NHSL2
**Yellow**	DCBLD2, DDX17, PFKP, CHSY1, NCS1, BDNF, PACSIN2, CMIP, MAP3K4, TMEM87A, RAP1GAP2, MLLT11, SELPLG, FLRT3

Table S2: High connectivity hub genes in each module.

Module	Final hub genes	Enriched biological process (TOP 10)
**Red**	GPAM, PNPLA2,<br>DLAT, ACACB,<br>ADIPOQ, ACSL5,<br>LIPE, LPL, PLIN1,<br>GPD1, FABP4, CD36,<br>PCK1,<br>PPARG,DGAT2	Metabolism of lipids<br>PPAR signaling pathway<br>Lipid localization<br>Regulation of lipid metabolic process<br>Lipid catabolic process<br>Generation of precursor metabolites and energy<br>Triglyceride metabolic process<br>Response to fatty acid<br>Primary alcohol metabolic process<br>AMPK signaling pathway
**Green**	SLC7A5, MYLIP,<br>DLX5	TNF signaling pathway<br>NF-kappa B signaling pathway<br>Interleukin-10 signaling<br>Positive regulation of cytokine production<br>Negative regulation of cytokine production<br>Small cell lung cancer<br>Response to osmotic stress<br>Negative regulation of immune system process<br>Maintenance of permeability of blood-brain barrier<br>Negative regulation of cell proliferation
**Tan**	MYOZ2, KLHL30	Muscle system process<br>Artery development<br>Cell morphogenesis involved in differentiation<br>Divalent inorganic cation homeostasis<br>Muscle cell differentiation<br>Glomerulus development<br>Regulation of ion transport<br>Central nervous system neuron differentiation<br>Extracellular matrix organization<br>Modulation of excitatory postsynaptic potential
**Grey60**	BIRC3, PTGS2	Regulation of neuron differentiation<br>Response to growth factor<br>Negative regulation of growth<br>Metallothioneins bind metals<br>Protein autophosphorylation<br>Signaling pathways regulating pluripotency of stem cells<br>Negative regulation of cAMP-mediated signaling<br>Circadian rhythm<br>PID LKB1 PATHWAY<br>Response to inorganic substance
**Yellow**	BDNF, SELPLG	Ribosome biogenesis<br>Flavonoid glucuronidation<br>Ribosomal small subunit biogenesis<br>Ribosomal large subunit biogenesis<br>RNA localization<br>Transcription by RNA polymerase I<br>Ribonucleoprotein complex assembly<br>mRNA processing<br>Nucleolus organization<br>RNA 3'-end processing

Table S3: Final hub genes and biological process in each module

2. Supplementary digital Data

Supplemental digital content 1: red module motifs enrichment

Supplemental digital content 2: green module motifs enrichment

Supplemental digital content 3: tan module motifs enrichment

Supplemental digital content 4: grey60 module motifs enrichment

Supplemental digital content 5: yellow module motifs enrichment

3. Supplementary Figures

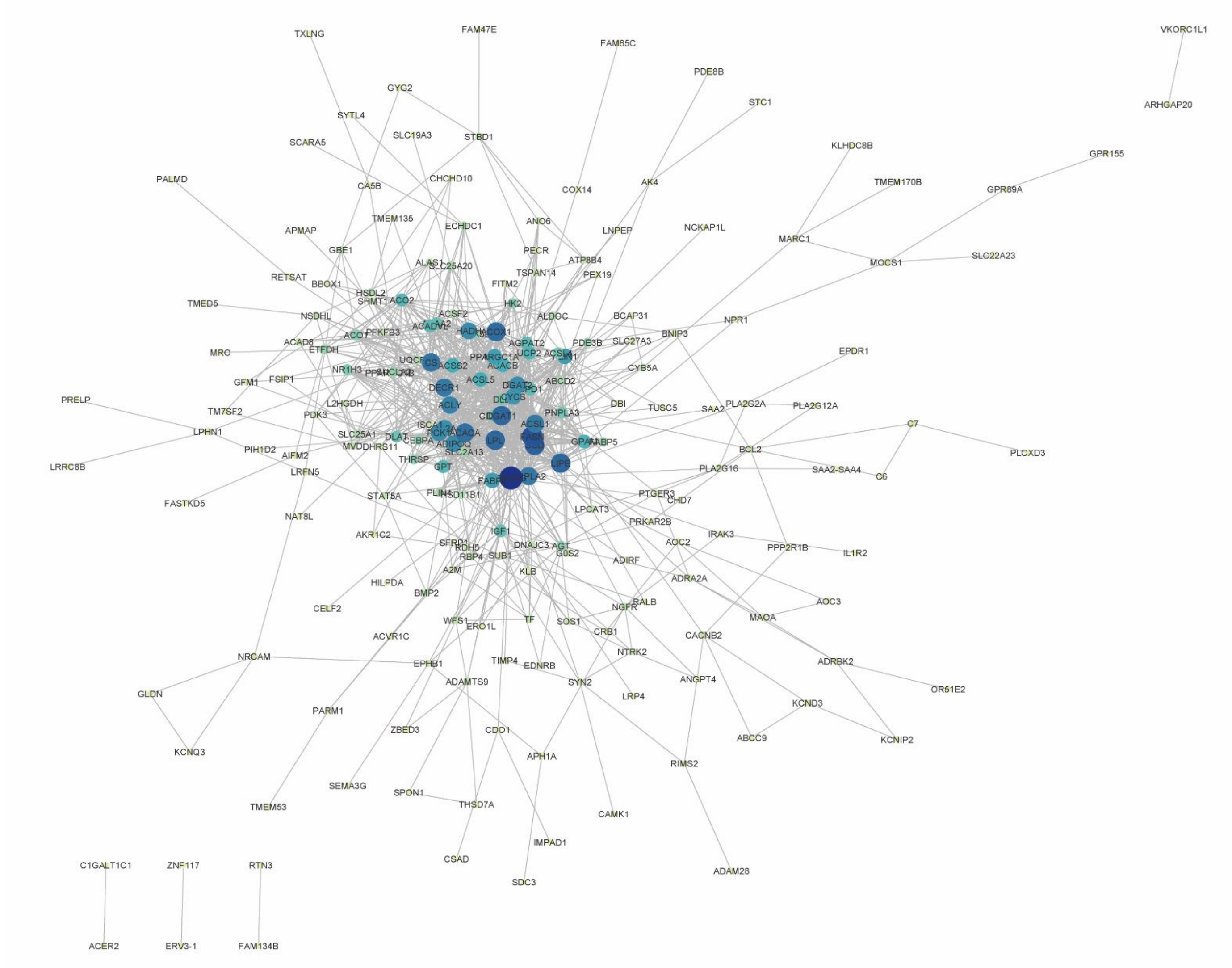

Figure S1A: Protein-Protein Interaction (PPI) network analysis for module red. The size of nodes and the shade of colour indicate the connectivity of each protein in the network.

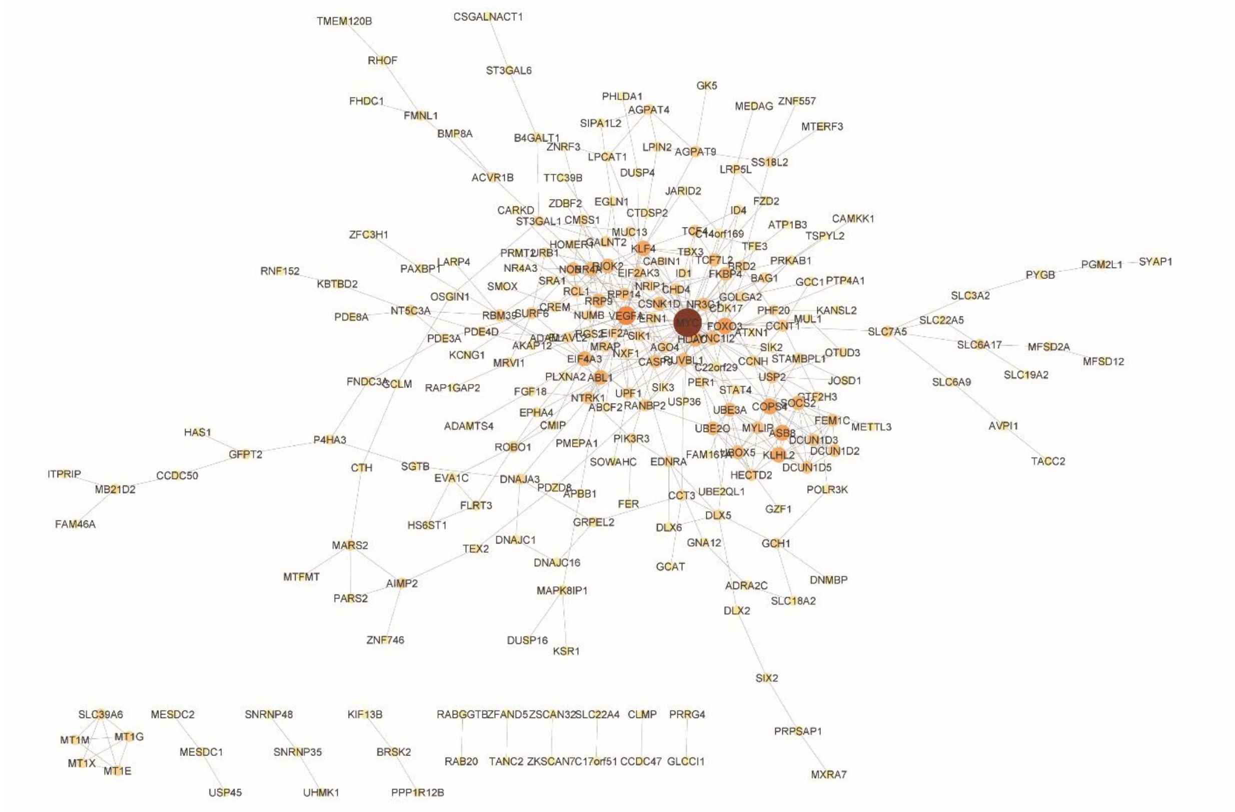

Figure S1B: Protein-Protein Interaction (PPI) network analysis for module green. The size of nodes and the shade of colour indicate the connectivity of each protein in the network.

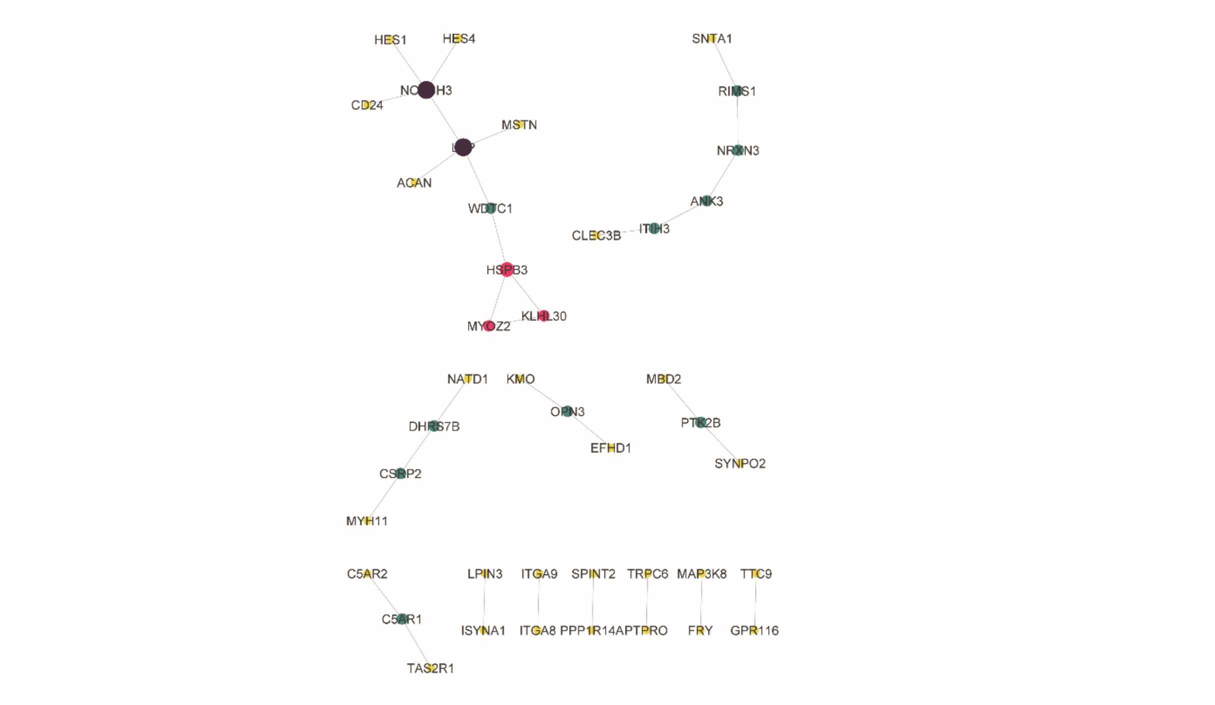

Figure S1C: Protein-Protein Interaction (PPI) network analysis for module tan. The size of nodes and the shade of colour indicate the connectivity of each protein in the network.

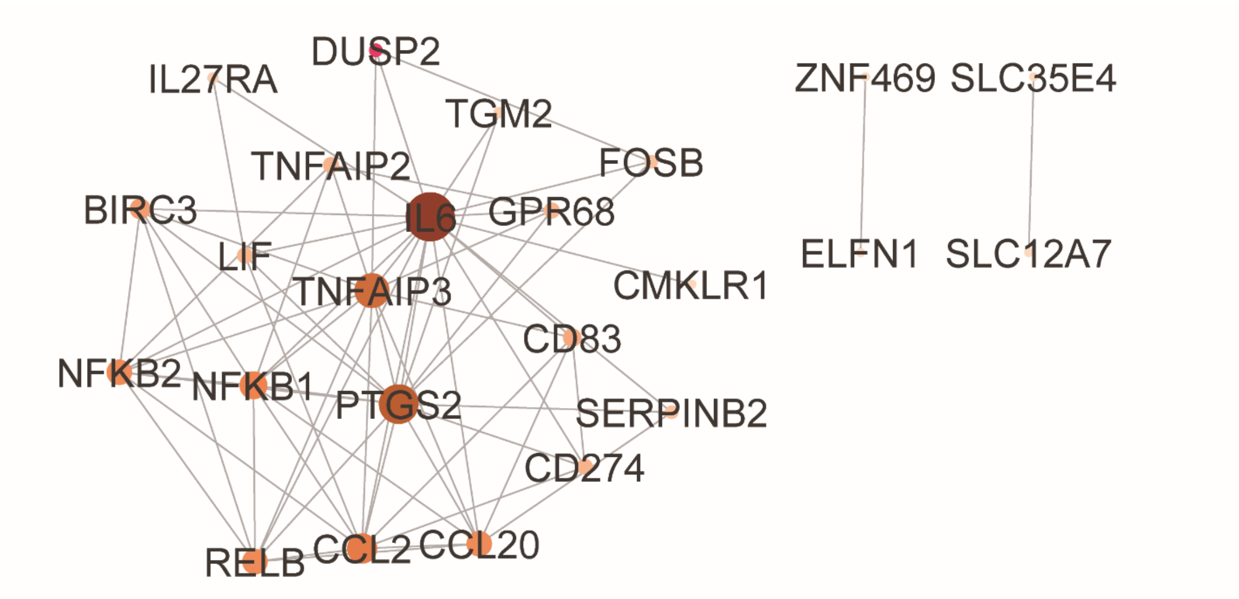

Figure S1D: Protein-Protein Interaction (PPI) network analysis for module grey60. The size of nodes and the shade of colour indicate the connectivity of each protein in the network.

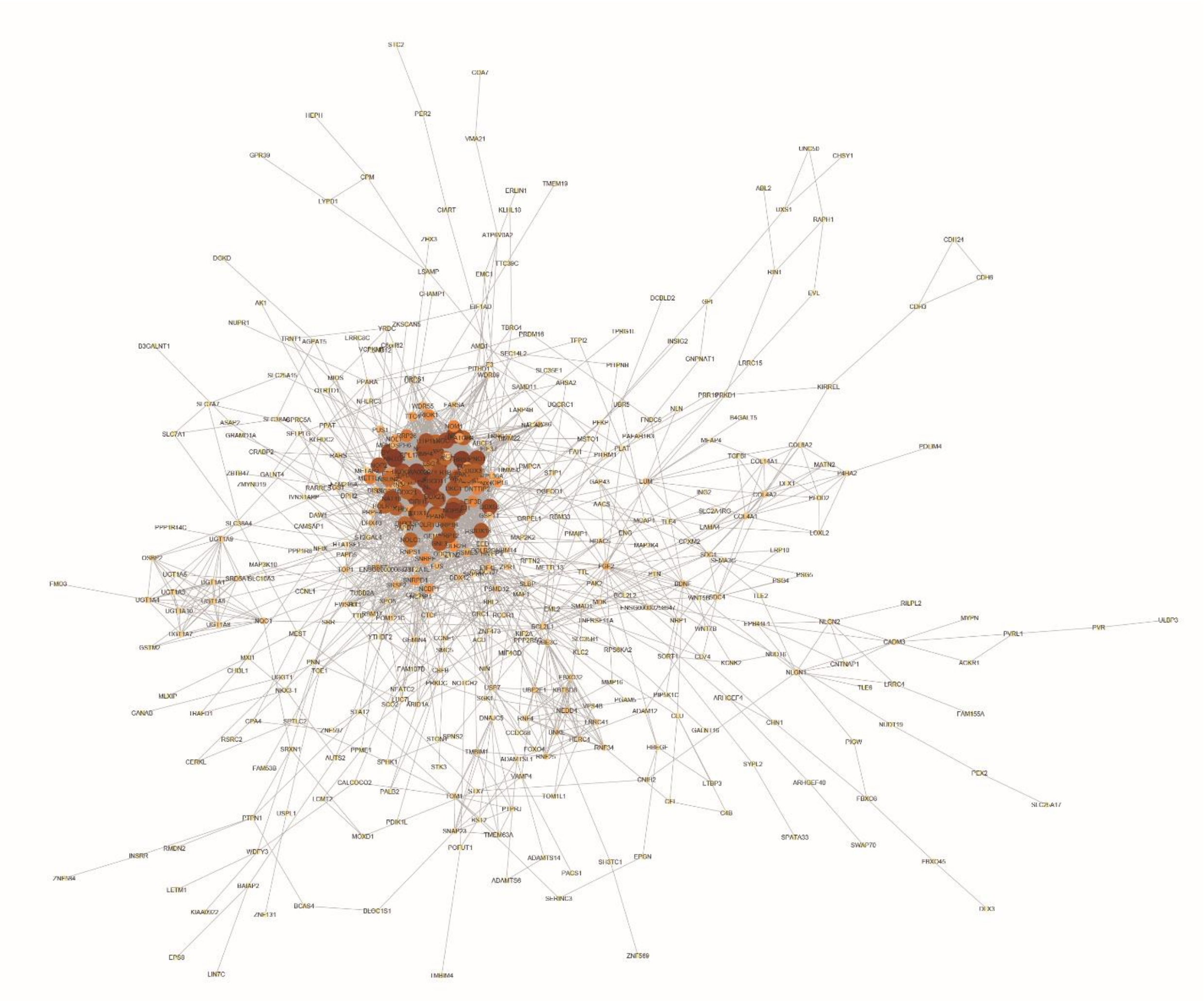

Figure S1E:Protein-Protein Interaction (PPI) network analysis for module yellow. The size of nodes and the shade of colour indicate the connectivity of each protein in the network.

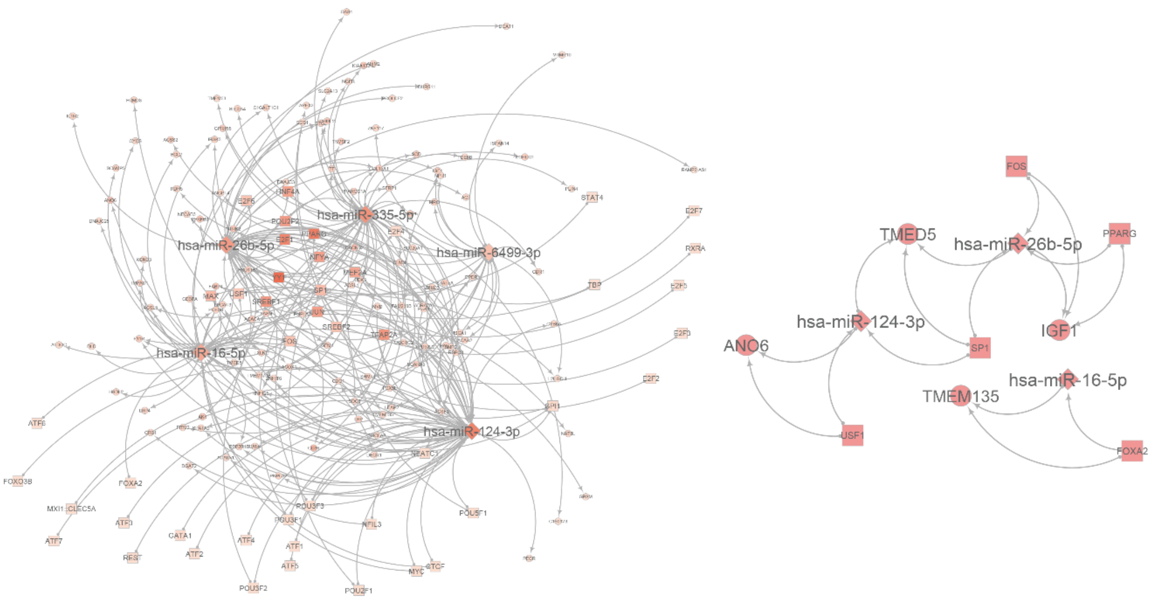

Figure S2A: miRNA-TF-mRNA network analysis for red module. Diamond, rectangle and ellipse represents miRNA, TF and mRNA, respectively. MiRNA-mRNA pairs which share the same TF are extracted from the network.

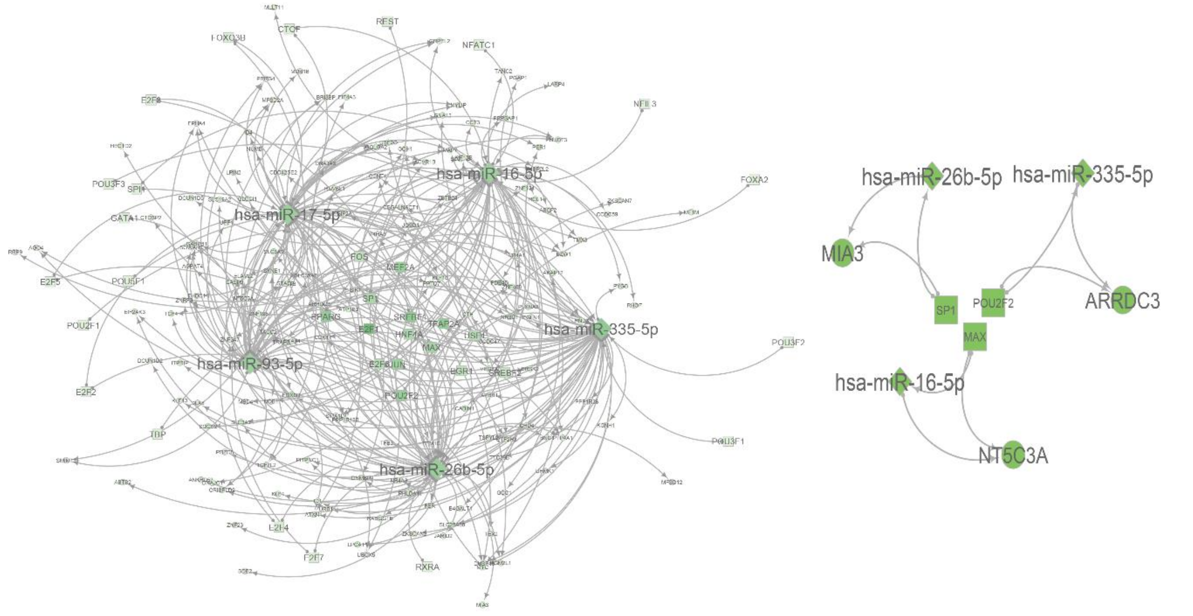

Figure S2B: miRNA-TF-mRNA network analysis for green module. Diamond, rectangle and ellipse represents miRNA, TF and mRNA, respectively. MiRNA-mRNA pairs which share the same TF are extracted from the network.

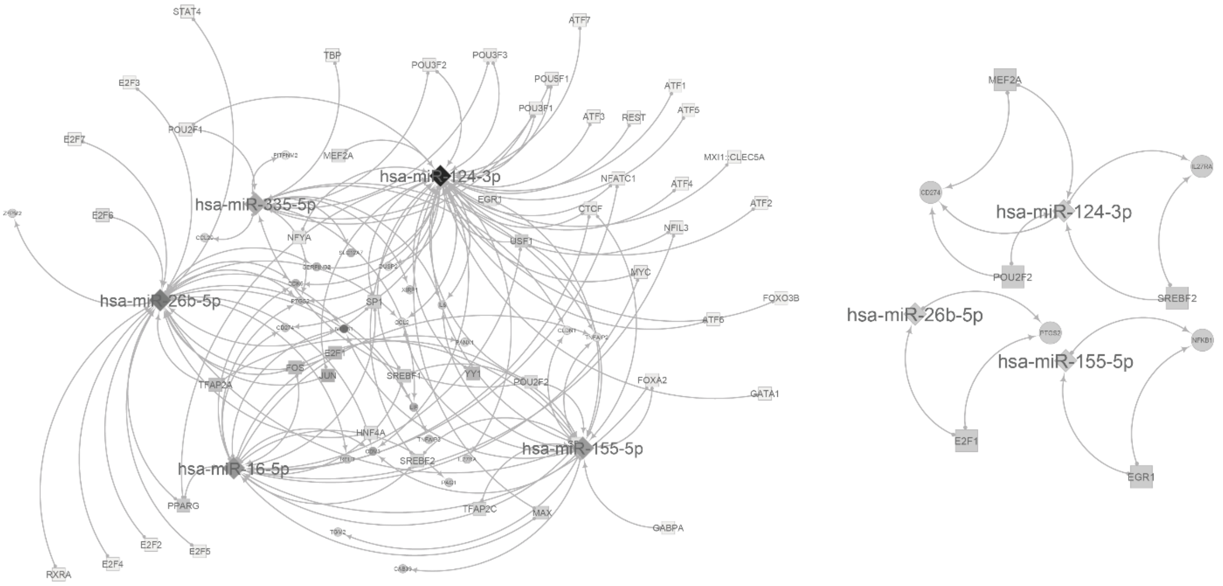

Figure S2C: miRNA-TF-mRNA network analysis for grey60 module. Diamond, rectangle and ellipse represents miRNA, TF and mRNA, respectively. MiRNA-mRNA pairs which share the same TF are extracted from the network.

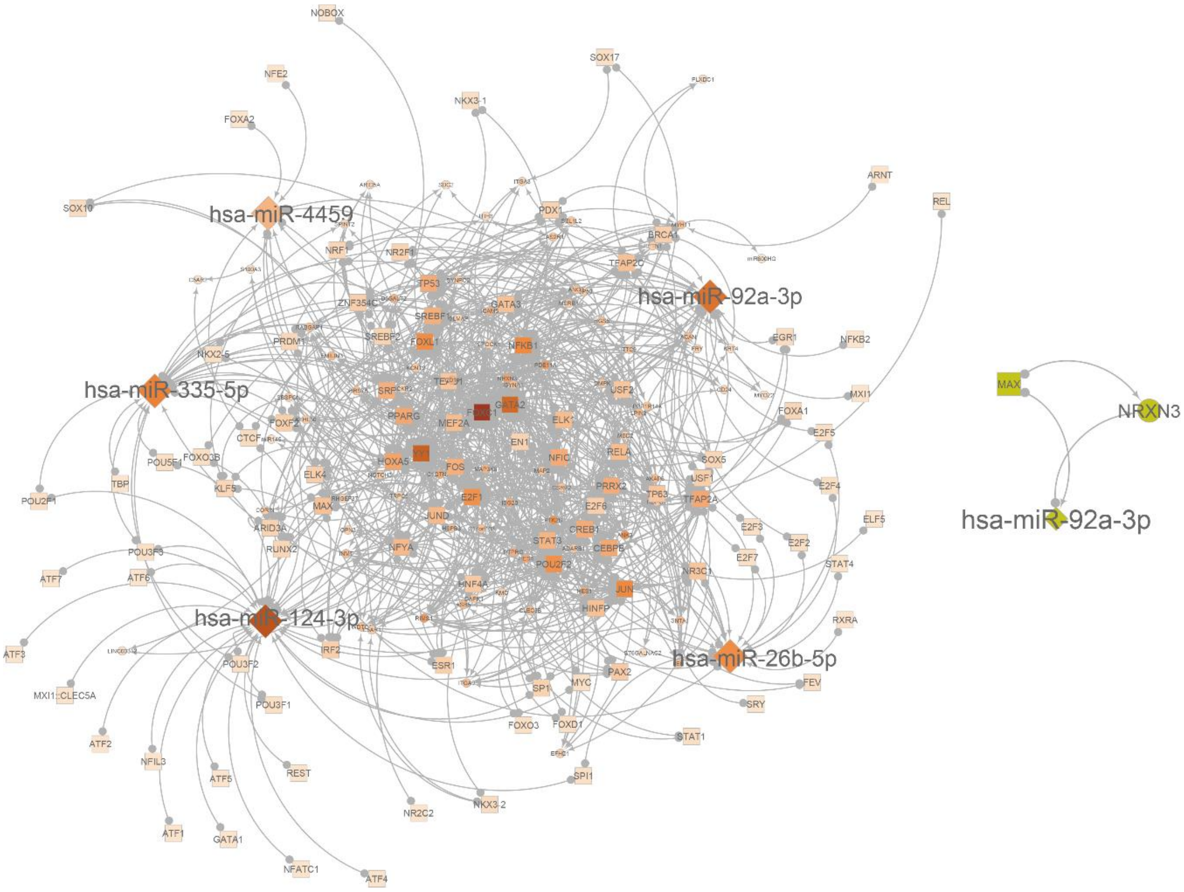

Figure S2D: miRNA-TF-mRNA network analysis for tan module. Diamond, rectangle and ellipse represents miRNA, TF and mRNA, respectively. MiRNA-mRNA pairs which share the same TF are extracted from the network.

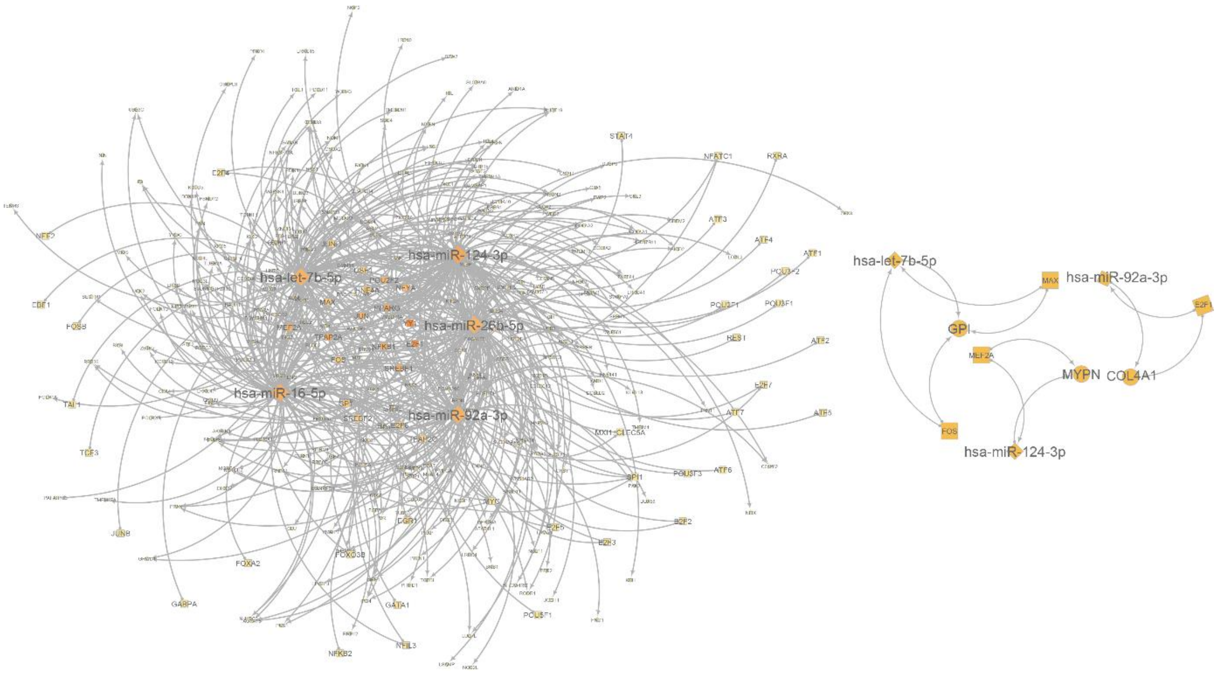

Figure S2E: miRNA-TF-mRNA network analysis for yellow module. Diamond, rectangle and ellipse represents miRNA, TF and mRNA, respectively. MiRNA-mRNA pairs which share the same TF are extracted from the network.

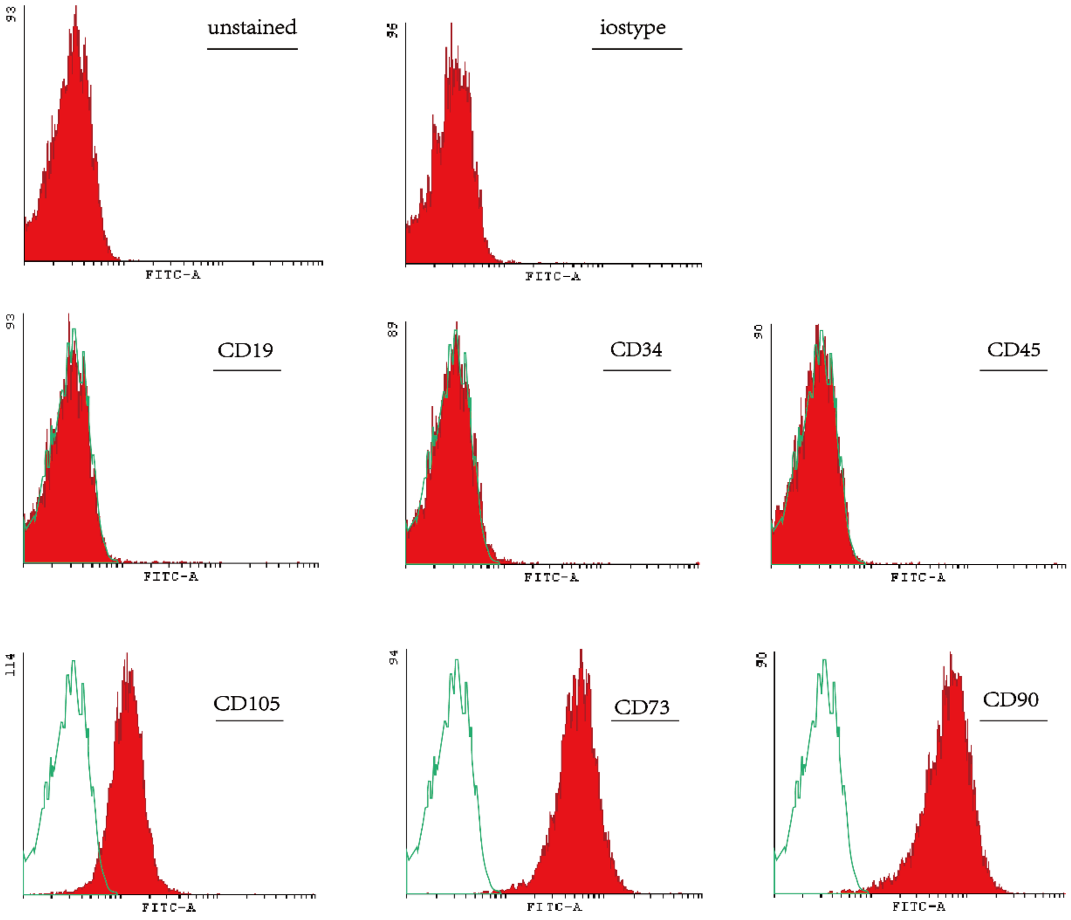

Figure S3: Immunophenotype characteristics of BMSC. BMSCs demonstrated immunophenotype characteristics of CD105+, CD73+, CD90+, CD19-, CD34- and CD45-. Unstained cell and isotype antibody are used as control.
